# To Bur off the End of Root

**Published:** 1904-06

**Authors:** Garrett Newkirk

**Affiliations:** Los Angeles.


### TO BUR OFF THE END OF ROOT.

By Garrett Newkirk, Los Angeles.

I seldom do more than make a small opening through which I can introduce a bur of suitable form for cutting off or smoothing the end of the root, or for breaking down any diseased bone. It is better, I think, not to make a large opening. All we need is a channel for washing out the debris, and then, when in an aseptic condition, the "pocket" will quickly close up.
—*Gazette.*

Apropos of the approaching jubilee of the University of Wisconsin, there is an article in the June *Review of Reviews*, by William B. Shaw, telling something of the work of that vigorous institution, as typical of the state universities that have come so rapidly to the front in recent years, especially in the middle west. The University of Wisconsin now has a larger student attendance than Yale's, a faculty of 250, and an annual income of $500,000.

